# Trends in High-Severity Billing of Hospitalized Medicare Beneficiaries Treated by Hospitalists vs Nonhospitalists

**DOI:** 10.1001/jamahealthforum.2022.0120

**Published:** 2022-03-18

**Authors:** Agustina D. Saenz, Yusuke Tsugawa, Jessica Phelan, E. John Orav, Jose F. Figueroa

**Affiliations:** 1 Department of Medicine, Brigham and Women’s Hospital, Harvard Medical School, Boston, Massachusetts; 2 Division of General Internal Medicine and Health Services Research, David Geffen School of Medicine at UCLA, Los Angeles, California; 3 Department of Health Policy and Management, UCLA Fielding School of Public Health, Los Angeles, California; 4 Department of Health Policy & Management, Harvard T.H. Chan School of Public Health, Boston, Massachusetts

## Abstract

**Importance:**

As US hospital expenditures continue to rise, understanding drivers of high-severity billing for hospitalized patients among inpatient physicians is critically important.

**Objective:**

To evaluate high-severity billing trends of Medicare beneficiaries treated by hospitalists vs nonhospitalists.

**Design, Setting, and Participants:**

This cohort study used Medicare fee-for-service claims of hospitalized patients from 2009 through 2018 to compare the proportion of high-severity billing between general medicine physicians classified as hospitalists vs nonhospitalists across initial, subsequent, and discharge hospital encounters. We compared physicians within the same hospital using hospital fixed effects and adjusted for patient demographics and comorbidities. Changes in the billing practices were assessed by investigating differences in slopes using an interaction term between physician type and time. Analyses were conducted between August 2021 and January 2022.

**Exposures:**

Treatment by hospitalists vs nonhospitalists.

**Main Outcomes and Measures:**

High-severity billing for initial, subsequent, and discharge hospital encounters.

**Results:**

The sample included 3 121 260 and 1 855 678 Medicare beneficiaries treated by hospitalists vs nonhospitalists, respectively. In each year, mean age, proportion female, proportion Black and Hispanic dual status, and mean number of chronic conditions were similar among those treated by hospitalists vs nonhospitalists (standardized mean difference < .01). The number of hospitalists grew by 76%, from 23 390 in 2009 to 41 084 in 2018, whereas nonhospitalists decreased by 43.6% (53 758 to 30 289). The proportion of encounters performed by hospitalists increased for the initial hospital encounters (46.3% to 76%), subsequent encounters (46.8% to 76.7%), and discharge encounters (46.1% to 78.5%) over the 10-year period. The proportion of high-severity billing across the hospital, subsequent, and discharge encounters was consistently higher among hospitalists relative to nonhospitalists across all years. Compared with the trends for nonhospitalists, the proportion of high-severity billing grew by 0.46% per year (95% CI, 0.44% to 0.49%; *P* < .001) for initial encounters, 0.38% per year (95% CI, 0.37% to 0.39%; *P* < .001) for subsequent encounters, and by 1.1% per year (95% CI, 1.1% to 1.15%; *P* < .001) for discharge encounters among hospitalists.

**Conclusions and Relevance:**

In this cohort study of Medicare fee-for-service beneficiaries treated in hospitals, high-severity billing increased over time for hospital encounters at higher rates for hospitalists than for nonhospitalists. These differences do not appear to be explained by patient complexity. The increase in the number of hospitalists over time may be contributing to rising national costs related to hospital care.

## Key Points

**Question:**

Are there substantial differences in high-severity billing of hospital encounters, defined as coding the highest level of severity, for Medicare patients treated by hospitalists vs nonhospitalists, and how are these differences changing over time?

**Findings:**

In this cohort study of 4 071 241 Medicare fee-for-service beneficiaries treated in hospitals between 2009 and 2018, hospitalists billed a significantly higher proportion of their initial, subsequent, and discharge encounters of Medicare beneficiaries as high-severity compared with nonhospitalists, even after accounting for patient complexity, and this gap is growing over time.

**Meaning:**

The growing number of hospitalists that are increasingly caring for Medicare beneficiaries and billing for higher-severity encounters may be an important driver of rising hospital costs nationally.

## Teaser

This cohort study characterizes differences in high-severity billing of hospital encounters for patients with Medicare treated by hospitalists vs nonhospitalists, and how these differences change over time.

## Introduction

The US has the most expensive health care system globally.^1^ Although much of its health care expenditures are attributable to the high prices the country pays for individual health care services,^2,3,4^ understanding other key drivers behind its high spending is critically important. One potential area that deserves further exploration is billing practices of inpatient stays across US hospitals. Recently, the Office of Inspector General (OIG), which is a federal agency responsible for eliminating waste and abuse in the US health care system, released a report citing concern for a prepandemic trend in more expensive inpatient hospital stays in the Medicare program.^5^ Given that hospital spending represents close to a third of total US health care spending,^6^ a further look into specific billing practices by hospitals and physicians is warranted.

Of particular interest is physician-level factors associated with variation in the billing of patient severity across evaluation and management (E/M) encounters in the hospital setting. In the US, individual physicians are responsible for coding the initial admission evaluation, subsequent encounters during the hospital stay, and discharge encounters, which subsequently affect physician reimbursement. There is likely considerable individual-level discretion at what constitutes a high-severity E/M billing. One potential source of variation may be driven by the type of inpatient medical professional who bills for patient encounters.

In recent years, the number of hospitalists across US hospitals has been increasing. Hospitalists are physicians that predominantly work in US hospitals and only care for patients while they are in the inpatient setting. Recent claims have raised concerns that hospitalists may be in part contributing to higher levels of intensity billing over time through upcoding.^7^ However, there is little empirical evidence on this topic on a national scale. As the number of hospitalists continues to grow and hospitalists disproportionately care for general medicine admissions, it is important to determine the extent to which hospitalists may be driving trends in the billing of high-severity E/M visits in the hospital setting.

Therefore, using national Medicare claims data, we sought to answer 3 questions. First, what proportion of initial hospital E/M encounters, subsequent encounters, and discharge encounters are done by general medicine hospitalists vs nonhospitalists, and how is this changing over time? Second, are there substantial differences in high-severity billing of initial hospital E/M encounters, subsequent encounters, and discharge encounters by hospitalists vs nonhospitalists even after adjustment of patient complexity? Finally, are differences in high-severity E/M billing of hospital encounters changing over time between hospitalists and general medical services (GMS) physicians?

## Methods

### Data

We used a sample of Medicare Part A and B claims of Medicare fee-for-service beneficiaries from 2009 to 2018 (5% sample was used for 2009 to 2010 and 20% sample from 2011 to 2018). We limited analyses to hospital claims billed by hospitalist physicians and other GMS physicians. Hospitalists were defined as physicians with primary specialties in internal medicine, general practice, family practice, or hospitalist where 90% of their E/M claims within a given year were for inpatient services (Current Procedural Terminology [CPT] codes: 99221, 99222, 99223, 99231, 99232, 99233, 99251, 99252, 99253, 99254, 99255). The accuracy of this approach has been validated by calling physicians in prior work (sensitivity of 84.2%, specificity of 96.5%, and a positive predictive value of 88.9%).^8^ The remaining general medicine physicians with the same primary specialties were defined as general medicine nonhospitalists. As done in prior studies, only physicians with at least 20 claims in a year were included to minimize potential misclassification of physician type.^8,9^ Claims performed by other specialty clinicians were excluded. Therefore, this study focused only on encounters performed by general medicine physicians.

We obtained patient demographics from the Medicare Beneficiary Summary File. Each hospitalization was categorized by their Medicare Severity Diagnosis Related Group (MS-DRG). From each hospitalization, index comorbidities were classified using the Elixhauser comorbidity algorithm.^10^ We limited the number of comorbidities to the first 9 coded diagnoses to limit potential bias associated with changes in the number of allowable diagnoses able to be coded in 2011.^11^

This study followed the Strengthening the Reporting of Observational Studies in Epidemiology (STROBE) reporting guidelines. The study was approved by the Harvard T.H. Chan School of Public Health institutional review board, which waived the requirement for informed consent because of the inability to contact enrollees in deidentified claims data.

### Outcomes

Our primary outcomes were high-severity billing codes for patients admitted by a hospitalist or other GMS physicians across 3 encounters: initial hospital encounter, subsequent hospital encounters, and discharge encounters. Initial hospital encounters included high-severity CPT codes of 99223 vs low- or moderate severity codes (99221 and 99222). Subsequent encounters included a high-severity CPT code of 99233 vs low- or moderate-severity codes (99231 and 99232). Discharge encounters included a high-severity code for more than 30 minutes (99239) vs low severity for 30 minutes or fewer (99238). Of note, for each admitted patient, only 1 type of physician was able to bill for an initial hospital encounter or a discharge encounter. Subsequent encounter billing was limited to only 1 claim per day by 1 physician.

### Statistical Analyses

We first compared the characteristics of patients admitted by hospitalists vs nonhospitalists in 2011 (the first year of the sample) and in 2018 (the last year available for analysis in this study). We used standardized mean differences (SMD) to compare key demographics and differences in comorbidities, of which those less than 0.10 were considered comparable.

Next, we used a multivariable linear regression model with the patient as the unit of analysis and whether high-severity billing occurred during the initial hospital encounter as the primary outcome. The primary exposure variable was the type of physician (hospitalist vs nonhospitalist), year (as a continuous variable), and the interaction term between physician type and year. The significance of the interaction term determines whether trends in high-severity billing differed over time between hospitalists vs nonhospitalists. Models included covariates for age, sex, dual status, race and ethnicity (Black, Hispanic, White, and Other), Elixhauser comorbidities, and MS-DRGs (to adjust for the primary reason for hospitalization). Hospital fixed effects were included to account for correlation in the hospital over time. Identical analyses were run for high-severity billing of subsequent encounters and discharge encounters. We repeated the models above using year as a categorical variable to obtain yearly point estimates of high-severity billing across the different types of encounters. Two-tailed *t* tests were considered significant at the Bonferroni adjusted level of *P* < .0167, to account for testing differences between trends across 3 different encounter outcomes. Analyses were performed using SAS statistical software (version 9.4, SAS Institute).

### Sensitivity Analyses

We performed several additional sensitivity analyses for robustness checks. First, we repeated our main models using the CMS Chronic Condition Data Warehouse comorbidities instead of Elixhauser comorbidities given that it is possible that some chronic conditions may not be coded during initial hospitalization. Next, we repeated our main models by further adjusting for hospital discharge destination in case there were other important differences in complexity treated by physicians who were classified as hospitalists vs nonhospitalists. We also examined differences in high-severity coding of a group of physicians who were originally classified as a nonhospitalist though later became a hospitalist. For this analysis, we compared rates of high-severity coding while they were a nonhospitalist vs coding while they were a hospitalist. We also examined rates of high-severity coding after excluding people with any critical care codes (either 99291 or 99292) at any point in the hospitalization in case there were potential differences in the type of patient who was admitted to a hospitalist vs a nonhospitalist.

Finally, to explore the extent to which patient-level characteristics vs hospital-level characteristics vs physician type (hospitalist vs nonhospitalist) contributes to variability of high-severity coding across the 3 encounter types, we substituted random effects for fixed effects in our models. We then sequentially added patient characteristics, hospital characteristics, and an indicator for physician type (hospitalist vs nonhospitalist).

## Results

### Patient and Hospital Characteristics by Physician Type

**Figure 1.**
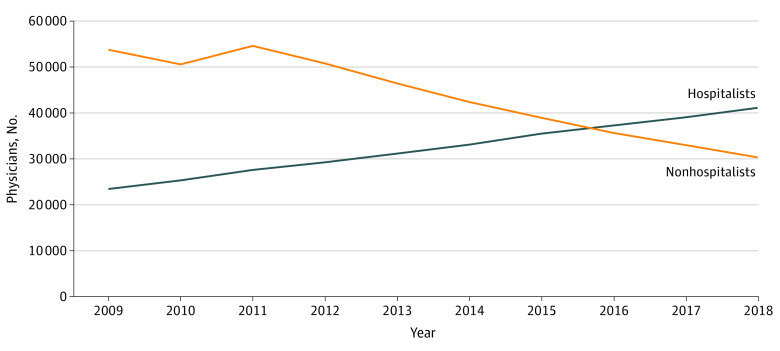
Number of General Medicine Hospitalists vs Nonhospitalists Treating Hospitalized Medicare Beneficiaries Over Time

**Figure 2.**
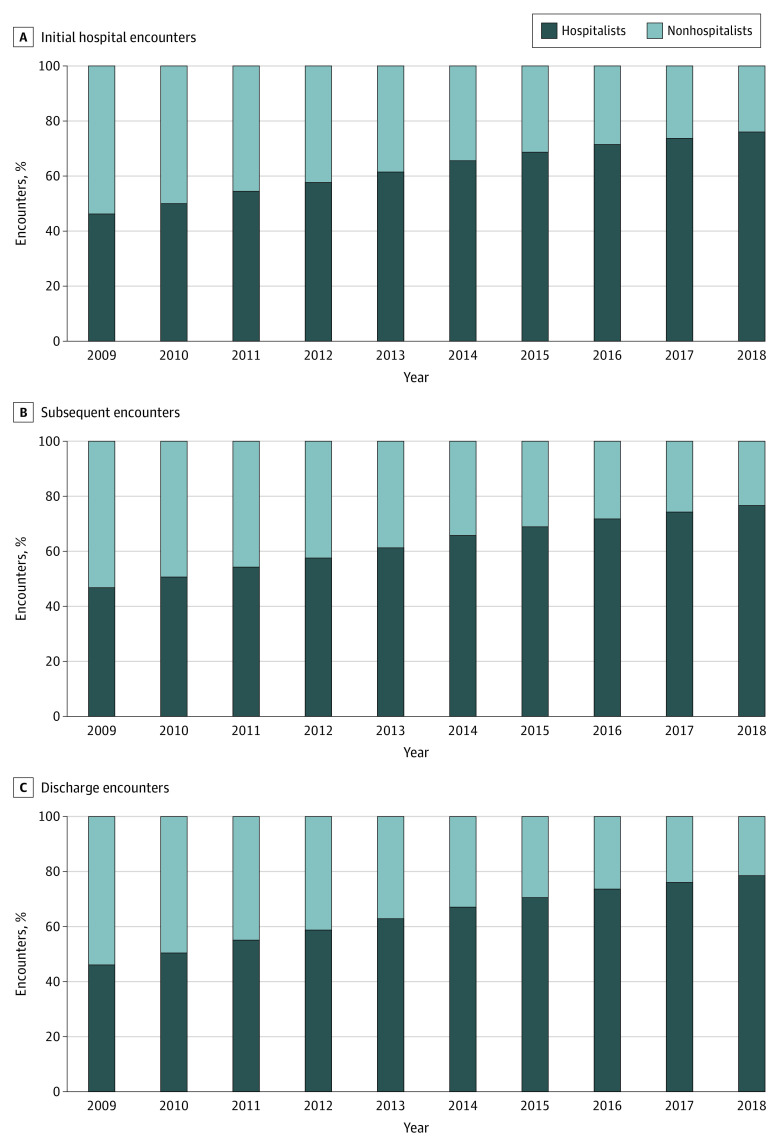
Proportion of Initial, Subsequent, and Discharge Encounters Performed by Hospitalists vs Nonhospitalists A, Proportion of initial hospital encounters performed by hospitalists vs nonhospitalists. B, Proportion of subsequent encounters performed by hospitalists vs nonhospitalists. C, Proportion of discharge encounters performed by hospitalists vs nonhospitalists

The number of physicians classified as hospitalists that treated hospitalized Medicare beneficiaries grew by 76%, from 23 390 in 2009 to 41 084 in 2018 (Figure 1). For the nonhospitalists, the trend was reversed, with the number of nonhospitalists billing at least 1 hospital encounter decreasing by 43.7% from 53 758 in 2009 to 30 289 in 2018. The proportion of total inpatient encounters performed by hospitalists increased for the initial hospital encounters (46.3% to 76%), subsequent encounters (46.8% to 76.7%), and discharge encounters (46.1% to 78.5%) over the 10-year period (Figure 2).

In 2009, the mean (SD) age of patients treated by nonhospitalists vs hospitalists was 75.7 (13.1) years vs 74.5 (13.8) years, respectively (SMD = 0.09) (Table 1). The proportion of female patients treated by nonhospitalists vs hospitalists was similar (59.3% vs 57.2%, SMD = 0.04) in 2009. The proportion of patients by race and ethnicity, dual status, the mean number of comorbidities, and specific types of comorbidities was also similar across both physician groups (all SMD values were <0.10). Patterns were consistent in 2018 (Table 1). We also examined the top 10% of DRG codes billed across the study period and found that the rates billed by hospitalists vs nonhospitalists were similar (All SMD values <0.10) (eTable 1 in the Supplement). Finally, we examined the hospital characteristics of where patients were treated by physician type. Patients treated by hospitalists were more likely to be treated in large hospitals, teaching hospitals, and nonprofit hospitals, and less likely to be treated in critical access hospitals (Table 1).

**Table 1.** Characteristics of Patients Treated by Hospitalists vs Non-Hospitalists

Characteristics	2009			2018		
	%		SMD	%		SMD
	Nonhospitalist	Hospitalist		Nonhospitalist	Hospitalist	
Hospitalizations, No.	157 510	136 074		281 883	899 310	
Patient characteristics						
Age, mean (SD), y	75.8 (12.97)	74.6 (13.6)	0.09	74.9 (12.96)	74.5 (12.97)	0.03
Sex						
Female	59.5	57.4	0.04	55.8	54.6	0.02
Male	40.5	42.6		44.2	45.4	
Race and ethnicity						
Black	12.3	12.5	0.02	13.9	11.6	0.08
Hispanic	2.2	2.2		2.6	2.1	
White	83.1	82.6		80.0	83.0	
Other^a^	2.4	2.8		3.5	3.4	
Dual status	30.2	30.9	0.01	30.8	28.1	0.06
Chronic conditions						
Mean (SD)	2.4 (1.3)	2.4 (1.3)	0.02	2.5 (1.3)	2.5 (1.3)	0.02
Congestive heart failure	14.8	14.5	0.01	19.2	19.0	0.005
Hypertension	54.8	53.5	0.03	49.3	47.7	0.03
Chronic pulmonary disease	20.6	19.2	0.04	19.4	18.6	0.02
Diabetes (combined)	26.5	24.7	0.04	27.6	26.5	0.02
Renal failure	14.2	15.9	0.05	18.7	18.5	0.007
Liver disease	1.6	1.9	0.02	2.2	2.3	0.008
Psychoses and depression	11.1	10.5	0.02	8.8	8.6	0.005
Hospital characteristics						
Hospital size						
Small (<99 beds)	16.6	12.7	0.21	12.7	11.1	0.08
Medium (100-399 beds)	60.4	55.3		58.5	56.9	
Large (>400 beds)	23.0	31.9		28.8	32.0	
Teaching status						
Major	11.3	15.6	0.18	15.5	16.0	0.07
Minor	26.4	30.5		27.2	30.2	
Nonteaching	62.3	53.9		57.3	53.8	
Profit status						
For-profit	15.2	13.3	0.07	16.3	12.6	0.13
Nonprofit	71.8	74.9		70.2	76.1	
Government, nonfederal	13.0	11.7		13.5	11.3	
Presence of medical ICU	83.2	86.1	0.08	83.2	87.3	0.12
Urban location	92.7	94.8	0.09	95.3	97.0	0.09
Region						
Northeast	18.0	19.5	0.21	18.4	17.8	0.17
Midwest	30.2	22.1		28.2	22.2	
South	40.3	42.3		40.3	42.0	
West	11.6	16.2		13.1	18.0	
Critical access hospital	5.0	4.0	0.05	3.4	2.3	0.07

Abbreviations: ICU, intensive care unit; SMD, standardized mean differences.

^a^ Other racial and ethnic minority group members included Asian, North American Native, and beneficiaries of “unknown” or “other” race as reported by the Medicare beneficiary race variable from the Social Security Administration.

### Differences in High-Severity Billing

**Figure 3.**
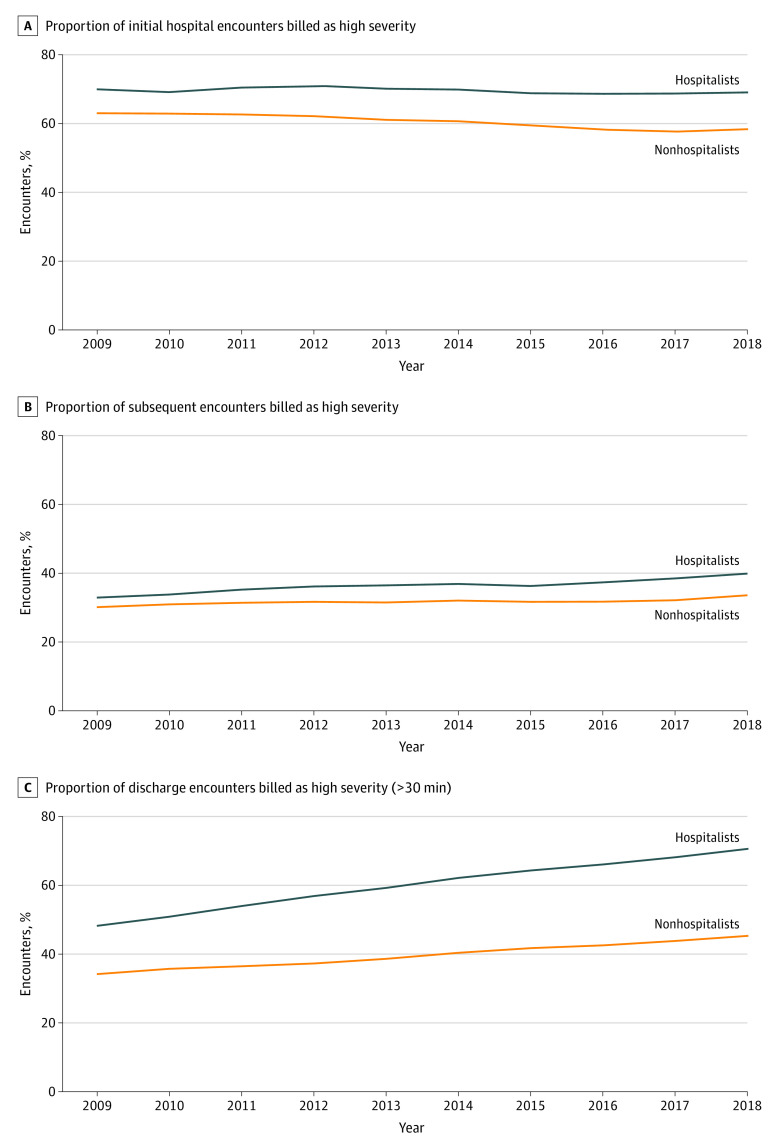
Trends of High Severity Billing of Hospital Encounters by Hospitalists vs Nonhospitalists A, Proportion of initial hospital encounters billed as high severity. B, Proportion of subsequent encounters billed as high severity. C, Proportion of discharge encounters billed as high severity (>30 minutes)

From 2009 through 2018, the proportion of billing of initial hospital encounters as high severity was higher across all yearly time points than the encounters billed by nonhospitalists, even after risk-adjustment for patient-level factors (Figure 3A). Over time, the proportion of initial hospital encounters coded as high severity remained fairly consistent for hospitalists but decreased slightly among nonhospitalists. In 2009, 69.9% of initial encounters by hospitalists were billed as high severity compared with 63.0% by nonhospitalists (6.9%, 95% CI, 6.6%-7.3%; *P* < .001) (Table 2). In 2018, 69.1% of initial encounters were billed as high severity by hospitalists compared with 58.4% by nonhospitalists (10.7%; 95% CI, 10.5% to 10.9%; *P* < .001). The yearly slope change for high-severity billing of initial encounters was −0.24% per year for hospitalists vs −0.71% per year for nonhospitalists (difference in yearly slopes of 0.46% per year; 95% CI, 0.44%-0.49%; *P* < .001).

**Table 2.** Differences in High-Severity Billing for Hospital Encounters Between Hospitalists vs Nonhospitalists

Type of encounter	%			Difference-in-slopes, % (95% CI)^b^	*P* value
	Baseline year (2009)^a^	Latest year (2018)^a^	Yearly change (slope)^b^		
Initial hospital encounters coded as high severity					
Hospitalists	69.9	69.1	−0.24	0.46 (0.44 to 0.49)	<.001
Nonhospitalists	63.0	58.4	−0.71		
Difference, % (95% CI)	6.9 (6.6 to 7.3)	10.7 (10.5 to 10.9)			
Subsequent hospital encounters coded as high severity					
Hospitalists	32.9	39.9	0.60	0.38 (0.37 to 0.39)	<.001
Nonhospitalists	30.1	33.6	0.22		
Difference, % (95% CI)	2.8 (2.6 to 3.0)	6.3 (6.2 to 6.4)			
Discharge encounters coded as high severity					
Hospitalists	48.2	70.6	2.37	1.12 (1.10 to 1.15)	<.001
Nonhospitalists	34.2	45.3	1.25		
Difference, % (95% CI)	14.0 (13.7 to 14.4)	25.3 (25.1 to 25.5)			

Abbreviation: MS-DRG, Medicare Severity Diagnosis Related Group.

^a^ Estimates were calculated using linear regression models treating year as a categorical predictor, adjusting for demographics, comorbidities, MS-DRGs, and with hospital fixed effects.

^b^ Estimates were calculated using linear regression models treating year as a continuous predictor to obtain slope, adjusting for demographics, comorbidities, MS-DRGs, and with hospital fixed effects.

Hospitalists billed higher severity for subsequent encounters compared with nonhospitalists across all years (Figure 3B). In 2009, hospitalists billed 32.9% of subsequent encounters as high severity compared with 30.1% of encounters by nonhospitalists (2.8% 95% CI, 2.6%-3.0%; *P* < .001) (Table 2). By 2018, the proportion of subsequent encounters billed as high severity increased more for hospitalists to 39.9% compared with nonhospitalists at 33.6% (6.3%; 95% CI, 6.2%-6.4%; *P* < .001). The yearly slope change for high severity billing was greater for hospitalists than nonhospitalists (difference in yearly slopes of 0.38% per year; 95% CI, 0.37%-0.39%; *P* < .001).

Across all time periods, hospitalists also billed higher for discharge encounters, and this gap increased over time (Figure 3C). In 2009, 48.2% of discharge encounters were billed as high severity (>30 minutes) compared with 34.4% by nonhospitalists (14.0%; 95% CI, 13.7%-14.4%; *P* < .001) (Table 2). By 2018, the proportion of discharge encounters billed as high severity increased for hospitalists to 70.6% compared with 45.3% for nonhospitalists, which corresponded to a difference of 25.3% (95% CI, 25.1%-25.5%; *P* < .001). The yearly change in slope was greater for hospitalists compared to nonhospitalists (difference in yearly slopes of 1.1% per year; 95% CI, 1.1%-1.15%; *P* < .001).

### Results of Sensitivity Analyses

We repeated our main models using the CCW algorithm instead of Elixhauser comorbidities, and our results were consistent (eTable 2 in the Supplement). Next, we further adjusted our models by adding a variable for discharge destination (unadjusted rates of discharge destination between hospitalists and nonhospitalists are shown in eTable 3 in the Supplement), and these results again yielded similar results as our main models (eTable 4 in the Supplement). We also repeated our models by excluding beneficiaries who received any critical care (as determined by critical care–level codes) during the hospitalization, and these results again were consistent with our main models where hospitalists coded higher severity compared wih nonhospitalists across all 3 encounter types (eTable 5 in the Supplement). Furthermore, in a subgroup of physicians who transitioned from being classified as nonhospitalist to hospitalist within the 10-year study period, we found that their rate of high-severity billing increased by 4.1% (95% CI, 3.8%-4.4%) for initial hospital encounters, 2.5% (95% CI, 2.3%-2.6%) for subsequent encounters, and 9.0% (95% CI, 8.7%-9.4%) for discharge encounters after they became a hospitalist compared with when they were a nonhospitalist (eTable 6 in the Supplement).

Finally, when examining the measured reasons for variability of high-severity coding (eTable 7 in the Supplement), the observed hospital characteristics explained the largest proportion of between-hospital variation for high-severity coding of Medicare beneficiaries across the 3 different types of encounters. Physician type (hospitalist vs nonhospitalist) explains a smaller proportion of the variation for initial hospital encounters and subsequent encounters, though it increased in the later years. For discharge encounters, physician type explains a much larger proportion of the variation in high-severity billing, which has increased as well in the later years, consistent with our main models.

## Discussion

Using national data on Medicare beneficiaries, we found that the number of hospitalists and their share of encounters in the hospital setting is growing over time. Hospitalists coded higher severity across the initial hospital, subsequent, and discharge encounters compared with nonhospitalist general medicine physicians. The gap in higher-severity billing between hospitalists and nonhospitalists has significantly grown, particularly for subsequent and discharge encounters. The differences in the observed complexity of patients being treated by hospitalists vs nonhospitalists do not seem to explain the differences or widening gap in billing patterns between the groups. Taken together, our results suggest that part of the increased hospital expenditures in the Medicare program may be in part driven by an increase in the number of hospitalists caring for Medicare beneficiaries and their associated higher-severity billing.

Drivers behind higher coding intensity among hospitalists are potentially multifactorial. First, it is important to note that higher patient complexity does not seem to justify why hospitalists are billing higher intensity across time relative to nonhospitalists in a given hospital, or why this gap across the 3 different types of encounters is growing, especially for discharge encounters. Patient demographics, dual status, race and ethnicity, and comorbidity burden were quite similar across both physician groups. Discharge destination posthospitalization was also quite similar across both groups. It is possible that some of the differences may be explained by the fact that some primary care physicians (PCPs) admit their own patients to hospitals. In these situations, PCPs likely know their patients quite well and thus may be more efficient at writing their notes, reading their medical charts, examining and speaking with patients, and preparing their discharge paperwork. This could translate to less time spent for each encounter, which is a key component in the E/M criteria that determines the level of billing. The extent to which this is occurring is unfortunately not assessable in this work, as we lacked detailed information about whether inpatient nonhospitalists were, in fact, patients’ PCPs.

Another potential reason could be that hospitalists may be more likely to receive productivity incentives, which could translate to higher levels of compensation or bonuses based on the number of relative value units (RVUs) they bill. A 2018 survey from the American Medical Association showed that from 2012 to 2018, there was an increase in the percentage of internal medicine physicians that received compensations predominantly based on personal productivity (RVUs).^12,13^ Therefore, it is possible that increases in personal productivity compensation models for hospitalists led to either more accurate vs more aggressive coding (and potentially inappropriate upcoding) of hospital encounters. Currently, there is no national, detailed data on physician compensation and incentive schemes available for public research, but further work in this area is warranted. It is also possible that nonhospitalists, who spend much less time caring for patients in the hospital setting, may be undercoding for patient severity, which then results in the gap in billing. On the other hand, hospitalists may potentially devote more time and training to documentation practices that support higher-severity billing given that their entire source of billing revenue may come from treating hospitalized patients.

One notable trend was the significant increase in discharge encounters coded as greater than 30 minutes across both hospitalists and nonhospitalists, although a greater increase was observed among hospitalists. Over the past decade, several national health reform efforts have increased the accountability of hospitals and their physicians for postdischarge care and outcomes. For example, in 2011, the Centers for Medicare & Medicaid Services introduced the Hospital Readmissions Reduction Program, which penalizes hospitals for higher-than-expected readmission rates across common medical conditions and a subset of surgical conditions. Prior work has shown that this policy had a substantial effect on the practices of hospitals, including investment in care coordination and postdischarge strategies.^14^ Inpatient physicians are thus seeing increased pressure from leadership to improve transitions of care. This may then translate into spending more time communicating with PCPs, providing patient or caregiver education, and performing better medication reconciliation, all of which could account for the increase in higher-intensity billing for discharge encounters.^15^

Higher intensity being coded by hospitalists is concerning, especially given that prior data have suggested that nonhospitalist PCPs may have lower mortality rates while having similar readmission rates when caring for their patients in the hospital.^16,17^ The strength of our work is that we analyzed trends within hospitals by applying hospital fixed effects, which therefore compared billing practices of hospitalists vs nonhospitalists of patients treated in the same hospital. We believe that such a comparison is necessary given that hospitalists tend to practice more in large hospitals, academic hospitals, and urban settings, which may influence the average type and complexity of patients they care for relative to nonhospitalists.

Looking forward, CMS is proposing new rules to the physician fee schedule for 2022, for example, accounting for split/shared E/M visits with nonphysician practitioners, which may allow the clinician who spends the highest proportion of time to bill for the encounter.^18^ It is unclear how these changes will affect billing severity. However, as CMS continues to update billing codes, and if productivity continues to be a major source of inpatient physicians’ compensation, further investigations of potential upcoding is necessary to mitigate this.

Our work adds to a growing body of literature that examines trends in the billing of E/M encounters by physicians. In older work, there have been concerning trends of higher intensity billing between 2001 and 2010 across US hospitals.^19^ Our work expands on this using more contemporary data and compares billing patterns among hospitalists vs nonhospitalists. Prior work has also found evidence of higher intensity billing across other specialties, including emergency department physicians. A notable difference, however, is that patient complexity partially explained these trends,^20,21^ which is not likely to be the reason behind differences in hospitalists vs nonhospitalists’ billing patterns.

### Limitations

Our study has limitations. First, although we found that patients of hospitalists and nonhospitalists were similar in terms of demographics and comorbidities, it is still possible that patient populations may differ in unobservable ways. For example, it is possible that hospitalists may potentially receive sicker patients than nonhospitalists in a hospital. However, several sensitivity analyses, including models excluding patients with critical level care, models adjusting for discharge status, and models examining physicians who switched from being a nonhospitalist to a hospitalist, all support the finding that hospitalists bill higher-severity encounters compared with nonhospitalists. Second, our work is an observational study that cannot definitively conclude causal findings between physician specialty type and billing patterns. Third, we lacked key information on how physicians are compensated or whether RVU incentives exist, which could be a major driver behind higher-intensity billing across physician groups. Finally, these data included only Medicare beneficiaries, and therefore, our results may not be generalizable to non-Medicare populations.

## Conclusions

In this cohort study of Medicare fee-for-service beneficiaries treated in hospitals, we found that the proportion of high-severity billing was much higher for hospitalists than nonhospitalists in 2009 to 2018, and these gaps grew over time, especially for subsequent and discharge encounters. These differences do not appear to be explained by substantial differences in patient complexity of patients treated across US hospitals. The increases in hospitalists over time may be contributing to rising costs related to hospital care across the country. Future work in this area would be helpful to determine drivers of high-intensity billing, and importantly, develop strategies to mitigate potential upcoding.

## Notes

Supplement. eTable 1. Top 10 Diagnosis-Related Group (DRG) Codes Billed by Physician Type for Hospitalized Medicare Beneficiaries eTable 2. Differences in high-severity billing for hospital encounters between Hospitalists vs. non-Hospitalists (adjusting for CCW conditions) eTable 3. Discharge Destination of Hospitalized Beneficiaries Treated by Hospitalists vs. Non-Hospitalists eTable 4. Differences in high-severity billing for hospital discharge encounters between Hospitalists vs. non-Hospitalists (further adjusting for discharge status) eTable 5. Differences in high-severity billing for hospital encounters between Hospitalists vs. Non-Hospitalists (excluding patients who received any critical care during hospitalization) eTable 6. High-Severity Coding Among A Group of Physicians Before and After Becoming a Hospitalist eTable 7. Variability of high-severity billing attributed to patient factors, hospital factors, vs. physician type (hospitalist vs. non-hospitalist)
