# Supplementary material for: Trends in High-Severity Billing of Hospitalized Medicare Beneficiaries Treated by Hospitalists vs Nonhospitalists
Source: JAMA Health Forum. 2022 Mar 18;3(3):e220120. doi: 10.1001/jamahealthforum.2022.0120 (PMC8933743; doi:10.1001/jamahealthforum.2022.0120)
Supplement: Supplement. — eTable 1. Top 10 Diagnosis-Related Group (DRG) Codes Billed by Physician Type for Hospitalized Medicare Beneficiaries eTable 2. Differences in high-severity billing for hospital encounters between Hospitalists vs. non-Hospitalists (adjusting for CCW conditions) eTable 3. Discharge Destination of Hospitalized Beneficiaries Treated by Hospitalists vs. Non-Hospitalists eTable 4. Differences in high-severity billing for hospital discharge encounters between Hospitalists vs. non-Hospitalists (further adjusting for discharge status) eTable 5. Differences in high-severity billing for hospital encounters between Hospitalists vs. Non-Hospitalists (excluding patients who received any critical care during hospitalization) eTable 6. High-Severity Coding Among A Group of Physicians Before and After Becoming a Hospitalist eTable 7. Variability of high-severity billing attributed to patient factors, hospital factors, vs. physician type (hospitalist vs. non-hospitalist) [file jamahealthforum-e220120-s001.pdf]

Supplemental Online Content

Saenz AD, Tsugawa Y, Phelan J, Orav EJ, Figueroa JF. Trends in high-severity billing of hospitalized Medicare beneficiaries treated by hospitalists vs nonhospitalists. *JAMA Health Forum*. 2022;3(3):e220120. doi:10.1001/jamahealthforum.2022.0120

eTable 1. Top 10 Diagnosis-Related Group (DRG) Codes Billed by Physician Type for Hospitalized Medicare Beneficiaries
eTable 2. Differences in high-severity billing for hospital encounters between Hospitalists vs. non-Hospitalists (adjusting for CCW conditions)
eTable 3. Discharge Destination of Hospitalized Beneficiaries Treated by Hospitalists vs. Non-Hospitalists
eTable 4. Differences in high-severity billing for hospital discharge encounters between Hospitalists vs. non-Hospitalists (further adjusting for discharge status)
eTable 5. Differences in high-severity billing for hospital encounters between Hospitalists vs. Non-Hospitalists (excluding patients who received any critical care during hospitalization)
eTable 6. High-Severity Coding Among A Group of Physicians Before and After Becoming a Hospitalist
eTable 7. Variability of high-severity billing attributed to patient factors, hospital factors, vs. physician type (hospitalist vs. non-hospitalist)

This supplemental material has been provided by the authors to give readers additional information about their work.

© 2022 Saenz AD et al. *JAMA Health Forum.*

Appendix Table 1. Top 10 Diagnosis-Related Group (DRG) Codes Billed by Physician Type for Hospitalized Medicare Beneficiaries

Top 10 Diagnosis-Related Group (DRG) Codes of Hospital Admissions Across Study Period	2009			2018		
	Non-Hospitalist	Hospitalist	SMD	Non-Hospitalist	Hospitalist	SMD
871—septicemia or severe sepsis without mechanical ventilation >96 hours with major complication or comorbidity	3.0%	4.0%	0.050	6.8%	8.1%	0.051
470—major joint replacements or reattachment of lower extremity	1.3%	1.1%	0.016	3.8%	2.8%	0.055
291—heart failure and shock with major complication or comorbidity	2.7%	2.8%	0.007	4.8%	4.9%	0.007
391—esophagitis, gastroenteritis, and miscellaneous digestive disorders with major complication or comorbidity	2.9%	2.5%	0.027	2.0%	1.7%	0.016
690—kidney & urinary track infections without major complication or comorbidity	2.7%	2.3%	0.025	2.1%	1.7%	0.029
194—simple pneumonia with pleurisy with complication or comorbidity	2.6%	2.3%	0.021	2.1%	1.6%	0.036
292—heart failure and shock with complication or comorbidity	2.4%	2.0%	0.028	1.3%	1.1%	0.014
190—chronic obstructive pulmonary disease with major complication or comorbidity	2.0%	1.8%	0.011	2.0%	1.7%	0.018
193—simple pneumonia and pleurisy with major complication or comorbidity	1.7%	1.9%	0.016	2.2%	2.2%	0.003
872—septicemia or severe sepsis without mechanical ventilation >96 hours without major complication or comorbidity	0.9%	0.9%	0.003	1.8%	2.2%	0.033

© 2022 Saenz AD et al. *JAMA Health Forum.*

Appendix Table 2. Differences in high-severity billing for hospital encounters between Hospitalists vs. non-Hospitalists (adjusting for CCW conditions), years 2011 to 2018

Type of Encounter	Baseline Year (2011)	Latest Year (2018)	Yearly Change (Slope)	Difference-in-slopes [95% CI, p-value]
**Initial hospital encounters coded as high severity**				
Hospitalists	70.6%	69.1%	-0.31%	0.5% (0.4% - 0.5%) p=<.001
Non-Hospitalists	62.8%	58.4%	-0.78%	
Difference	7.8% (7.6% - 7.9%)	10.7% (10.6% - 10.9%)		
**Subsequent hospital encounters coded as high severity**				
Hospitalists	35.3%	40.0%	0.57%	0.4% (0.4% - 0.4%) p=<.001
Non-Hospitalists	31.5%	33.7%	0.19%	
Difference	3.8% (3.7% - 3.9%)	6.3% (6.2% - 6.4%)		
**Discharge encounters coded as high severity**				
Hospitalists	57.4%	73.9%	2.30%	1.0% (1.0% - 1.1%) p=<.001
Non-Hospitalists	39.9%	48.6%	1.26%	
Difference	17.5% (17.3% - 17.7%)	25.3% (25.1% - 25.6%)		

Note: We only had access to CCW data between 2011 to 2018 so models were limited to these years.

© 2022 Saenz AD et al. *JAMA Health Forum.*

Appendix Table 3. Discharge Destination of Hospitalized Beneficiaries Treated by Hospitalists vs. Non-Hospitalists

	2009	2010	2011	2012	2013	2014	2015	2016	2017	2018
**Non-Hospitalists**										
Discharge Location										
Discharged Home/Self-Care	47.5%	46.2%	45.5%	44.8%	43.7%	43.6%	42.8%	43.5%	43.1%	42.7%
Transferred to Another Hospital	2.2%	2.1%	2.0%	2.1%	2.1%	2.0%	2.0%	2.0%	2.0%	1.9%
Discharged to Skilled Nursing Facility	23.7%	23.9%	24.5%	24.4%	24.8%	24.8%	25.4%	24.4%	24.4%	24.6%
Discharged to Home/Home Health	15.3%	16.2%	15.9%	16.3%	16.7%	16.6%	16.5%	16.7%	17.3%	17.5%
Discharged to Hospice	2.2%	2.3%	2.5%	2.6%	2.8%	2.9%	3.1%	3.0%	3.1%	3.1%
Transferred to Inpatient Rehab	2.5%	2.5%	2.7%	2.9%	3.0%	3.2%	3.5%	3.6%	3.5%	3.6%
Transferred to LTC	1.1%	1.1%	1.3%	1.3%	1.4%	1.5%	1.3%	1.4%	1.4%	1.4%
Left against medical advice	0.3%	0.3%	0.3%	0.3%	0.3%	0.3%	0.3%	0.4%	0.4%	0.4%
Died in Hospital	1.6%	1.5%	1.6%	1.6%	1.5%	1.5%	1.5%	1.5%	1.4%	1.4%
Other Discharge Status	3.7%	3.8%	3.8%	3.7%	3.7%	3.7%	3.7%	3.5%	3.5%	3.4%
**Hospitalists**										
Discharge Location										
Discharged Home/Self-Care	46.5%	45.8%	46.0%	45.2%	44.3%	44.1%	43.1%	44.4%	44.0%	43.6%
Transferred to Another Hospital	1.9%	1.9%	1.8%	1.8%	1.8%	1.9%	1.9%	1.9%	1.9%	1.8%
Discharged to Skilled Nursing Facility	23.1%	23.3%	22.9%	23.3%	23.3%	23.4%	24.0%	22.7%	22.7%	22.8%
Discharged to Home/Home Health	16.4%	16.6%	16.7%	17.0%	17.7%	17.5%	17.6%	17.7%	18.5%	18.8%
Discharged to Hospice	2.7%	2.9%	3.1%	3.3%	3.5%	3.5%	3.7%	3.6%	3.7%	3.8%
Transferred to Inpatient Rehab	2.9%	3.0%	3.1%	3.2%	3.4%	3.6%	3.7%	3.7%	3.7%	3.7%
Transferred to LTC	1.2%	1.3%	1.2%	1.3%	1.3%	1.2%	1.1%	1.1%	1.0%	1.0%
Left against medical advice	0.3%	0.3%	0.3%	0.3%	0.3%	0.3%	0.3%	0.4%	0.4%	0.5%
Died in Hospital	1.5%	1.5%	1.4%	1.4%	1.5%	1.4%	1.4%	1.3%	1.3%	1.2%
Other Discharge Status	3.4%	3.4%	3.4%	3.2%	3.0%	3.0%	3.1%	3.1%	2.9%	2.8%

© 2022 Saenz AD et al. *JAMA Health Forum.*

Appendix Table 4. Differences in high-severity billing for hospital discharge encounters between Hospitalists vs. non-Hospitalists (further adjusting for discharge status)

Type of Encounter	Baseline Year (2011)	Latest Year (2018)	Yearly Change (Slope)	Difference-in-slopes [95% CI, p-value]
**Discharge encounters coded as high severity**				
Hospitalists	49.9%	72.8%	2.44%	1.2% (1.1% - 1.2%) p=<.001
Non-Hospitalists	36.0%	47.3%	1.27%	
Difference	13.9% (13.5% - 14.3%)	25.5% (25.3% - 25.8%)		

Appendix Table 5. Differences in high-severity billing for hospital encounters between Hospitalists vs. non-Hospitalists of patients who did not receive any critical care during hospitalization

Type of Encounter	Baseline Year (2011)	Latest Year (2018)	Yearly Change (Slope)	Difference-in-slopes [95% CI, p-value]
**Initial hospital encounters coded as high severity**				
Hospitalists	66.6%	65.7%	-0.27%	0.4% (0.4% - 0.5%) p=<.001
Non-Hospitalists	59.8%	55.2%	-0.71%	
Difference	6.8% (6.5% - 7.2%)	10.5% (10.3% - 10.7%)		
**Subsequent hospital encounters coded as high severity**				
Hospitalists	28.9%	36.0%	0.62%	0.4% (0.4% - 0.4%) p=<.001
Non-Hospitalists	26.7%	30.3%	0.25%	
Difference	2.2% (2.0% - 2.4%)	5.7% (5.6% - 5.8%)		
**Discharge encounters coded as high severity**				
Hospitalists	49.9%	72.8%	2.44%	1.2% (1.1% - 1.2%) p=<.001
Non-Hospitalists	36.0%	47.3%	1.27%	
Difference	13.9% (13.5% - 14.3%)	25.5% (25.3% - 25.8%)		

© 2022 Saenz AD et al. *JAMA Health Forum.*

Appendix Table 6. High-Severity Coding Among A Group of Physicians Before and After Becoming a Hospitalist

Type of Hospital Encounter	Proportion of Encounters Coded as High Severity When Physicians Were Classified as a Non-Hospitalist	Proportion of Encounters Coded as High Severity When Physicians Were Classified as a Non-Hospitalist	Differences in High-Severity Coding After Becoming a Hospitalist
Initial admission hospital encounter	55.6%	59.7%	4.1% (3.8% to 4.4%)
Subsequent hospital encounter	33.2%	35.7%	2.5% (2.3% to 2.6%)
Discharge encounter	51.8%	60.8%	9.0% (8.7% to 9.4%)

© 2022 Saenz AD et al. *JAMA Health Forum.*

Appendix Table 7. Variability of high-severity billing attributed to patient factors, hospital factors, vs. physician type (hospitalist vs. non-hospitalist)

	Initial Admission Encounter		Subsequent Hospital Encounters		Discharge Encounters	
**From Model 1**	Years 2009 to 2010	Years 2017 to 2018	Years 2009 to 2010	Years 2017 to 2018	Years 2009 to 2010	Years 2017 to 2018
Variability (random effect) in high-severity billing due to within-hospital factors	0.050	0.058	0.036	0.046	0.059	0.084
Variability (random effect) in high-severity billing due to between-hospital factors	0.198	0.192	0.186	0.207	0.198	0.171
**From Model 2**						
% of Within-hospital variability explained by patient characteristics	4.5%	8.0%	6.2%	8.1%	3.5%	3.6%
% of Between-hospital variability by patient characteristics	3.1%	4.8%	2.1%	2.3%	1.0%	0.7%
**From Model 3**						
% of Within-hospital variability explained by hospital characteristics	0%	0%	0%	0%	0%	0%
% of Between-hospital variability explained by hospital characteristics	22.9%	21.0%	15.9%	16.4%	11.2%	16.1%
**From Model 4**						
% of Within-hospital variability explained by physician type (hospitalist vs. non-hospitalist)	1.4%	2.7%	0.3%	1.1%	7.1%	10.0%
% of Between-hospital variability explained by physician type (hospitalist vs. non-hospitalist)	0.4%	0.8%	0.1%	0.2%	1.9%	4.4%

Note: For each time period, a series of 4 different models were performed:
Model 1: random effects model with only hospital random effects.
Model 2: random effects model with hospital random effects + patient characteristics (demographics and chronic conditions) to estimate the proportion of the observed variation of high-severity billing determined by patient characteristics within hospitals and also by the effect of serving varying patient populations between hospitals.
Model 3: random effects model with hospital random effects + patient characteristics + hospital characteristics (including teaching status, hospital size, region, rural vs. urban, ownership type, critical access hospital status, and whether it has a medical ICU) to estimate the proportion of the variation of high-severity billing between hospitals determined by hospital characteristics.
Model 4: random effects model with hospital random effects + patient characteristics + hospital type + physician type (hospitalist vs. non-hospitalist) to estimate the proportion of the variation of high-severity billing determined by physician type within hospitals and also by differences in the prevalence of hospitalists between hospitals.

© 2022 Saenz AD et al. *JAMA Health Forum.*
